# Choroid plexus and the blood–cerebrospinal fluid barrier in disease

**DOI:** 10.1186/s12987-020-00196-2

**Published:** 2020-05-06

**Authors:** Peter Solár, Alemeh Zamani, Lucie Kubíčková, Petr Dubový, Marek Joukal

**Affiliations:** 1 Department of Anatomy, Cellular and Molecular Neurobiology Research Group, Faculty of Medicine, Masaryk University, CZ-625 00 Brno, Czech Republic; 2 Department of Neurosurgery, Faculty of Medicine, Masaryk University and St. Anne´s University Hospital Brno, Pekařská 53, CZ-656 91 Brno, Czech Republic

**Keywords:** Choroid plexus, Blood–cerebrospinal fluid barrier, Inflammatory diseases, Neurodegenerative disease, Autoimmune disease, Stroke, Carcinoma

## Abstract

The choroid plexus (CP) forming the blood–cerebrospinal fluid (B-CSF) barrier is among the least studied structures of the central nervous system (CNS) despite its clinical importance. The CP is an epithelio-endothelial convolute comprising a highly vascularized stroma with fenestrated capillaries and a continuous lining of epithelial cells joined by apical tight junctions (TJs) that are crucial in forming the B-CSF barrier. Integrity of the CP is critical for maintaining brain homeostasis and B-CSF barrier permeability. Recent experimental and clinical research has uncovered the significance of the CP in the pathophysiology of various diseases affecting the CNS. The CP is involved in penetration of various pathogens into the CNS, as well as the development of neurodegenerative (e.g., Alzheimer´s disease) and autoimmune diseases (e.g., multiple sclerosis). Moreover, the CP was shown to be important for restoring brain homeostasis following stroke and trauma. In addition, new diagnostic methods and treatment of CP papilloma and carcinoma have recently been developed. This review describes and summarizes the current state of knowledge with regard to the roles of the CP and B-CSF barrier in the pathophysiology of various types of CNS diseases and sets up the foundation for further avenues of research.

## Background

The central nervous system (CNS) is protected against harmful substances contained in the blood by the blood–brain barrier (BBB) and the blood–cerebrospinal (B-CSF) barrier [1–3]. Even though the B-CSF barrier is more accessible than the BBB for many foreign invaders, the BBB has received more attention regarding CNS pathology. Nevertheless, there is a growing body of evidence showing that the B-CSF barrier plays a crucial role in the spread of inflammatory reactions from the periphery to the CNS and contributes to the pathogenesis and progression of various neurological disorders [4–7]. If the B-CSF barrier allows blood-borne pathogenic components to enter the CSF, they would contribute to neurotoxicity and neuronal dysfunction. On the other hand, reduction in the efflux of waste products (and the consequent presence of neurotoxic residues inside the brain), could interfere with neurotransmission, and lead to various disorders. The mechanism(s) by which a dysfunctioning B-CSF barrier contributes to pathogenesis is still poorly understood. Therefore, we aim to review recent knowledge of the changes and regulation of the B-CSF barrier in different diseases. A better understanding of the role played by the B-CSF barrier in CNS diseases can shed light on identifying therapeutic targets in neuropathology.

## Morphology and physiology of the B-CSF barrier

**Fig. 1:**
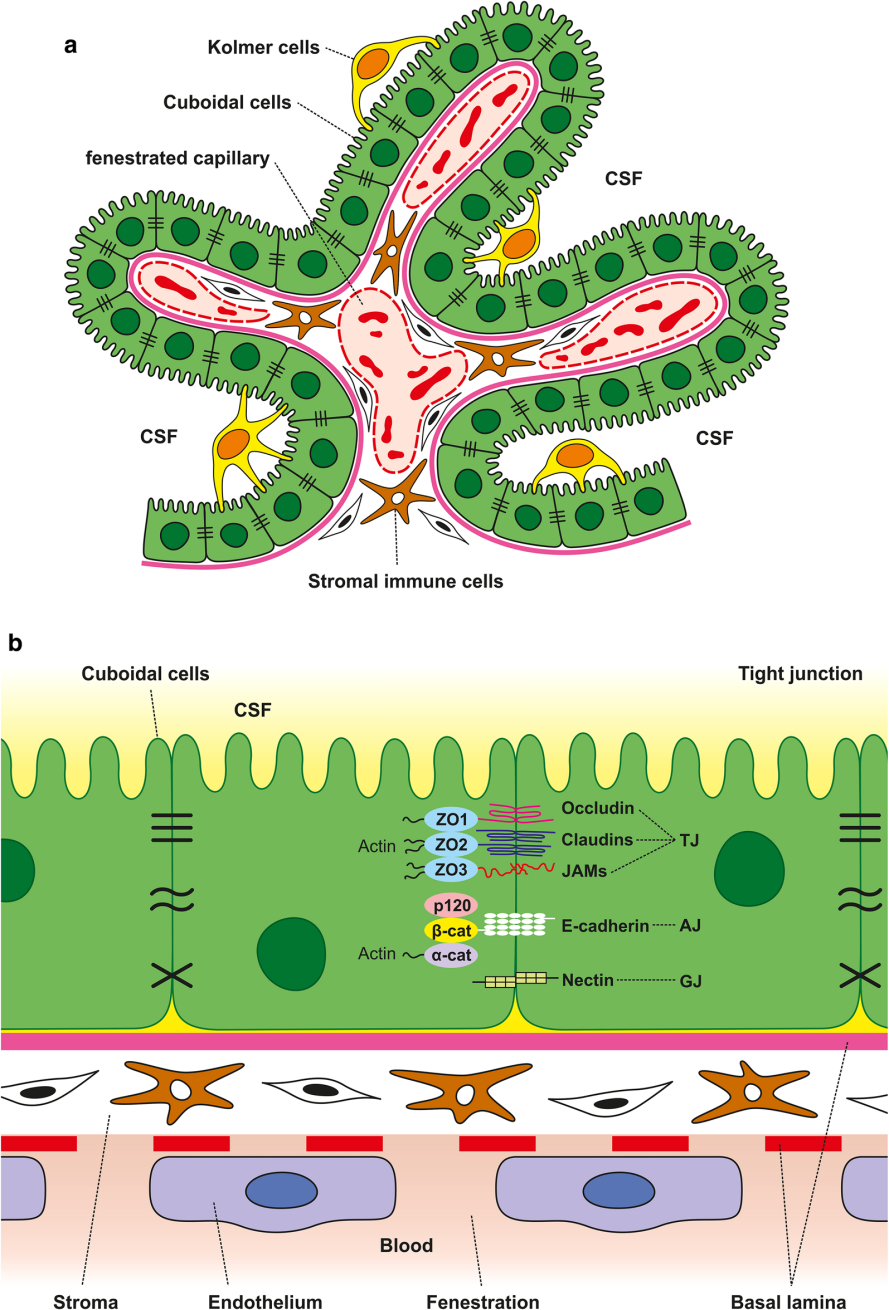
Schematic illustration of the anatomical organization of the CP (**a**) and the B-CSF barrier (**b**). The CP is an epithelio-endothelial convolute, comprising a highly vascularized stroma with connective tissue, and a continuous lining of epithelial cells with adhering Kolmer cells (**a**). The main site of the barrier (**b**) is at the level of the cuboidal epithelial cells that are linked by tight junctions (TJ), adherent junctions (AJ) and gap junctions (GJ). TJs are required for strong cell–cell adhesion and include transmembrane (occludin, claudins and JAMs) and cytoplasmic proteins (ZO). AJs are formed by E-cadherin, a transmembrane protein that intracellularly binds to actin through a variety of protein complexes, including catenin (p120, β-catenin, α-catenin). GJ protein complexes comprising nectin also play a role in adherence

The B-CSF barrier is localized in the choroid plexus (CP) of the brain ventricles. The CP is an epithelio-endothelial convolute, comprising a highly vascularized stroma with connective tissue, and epithelial cells [8–11]. The stroma comprises fenestrated capillaries with 60 to 80 nm fenestrations surrounded by connective tissue stroma with immune cells. The ventricular side of the stroma is covered by a single layer of cuboidal epithelial cells [10, 11]. Epiplexus (Kolmer) cells (KCs) with phagocytic activity adhere to the ventricular side of epithelial cells [12] (Fig. 1). The KCs communicate with epithelial cells of the through pannexin-1 channels and seem to be the scavengers of the brain ventricular system [13, 14]. The CP of the lateral ventricle is a thin undulating veil while in the fourth ventricle it is highly lobulated and more complex. The CP in the third ventricle is much smaller in size and has an intermediate appearance [10].

The primary function of the CP is to produce CSF and form the B-CSF barrier. Besides these roles, it has recently been described that the CP might be a part of the circadian regulatory system [15]. It has also been shown that the B-CSF barrier expresses chemosensory receptors such as odorant receptors, vomeronasal receptors, and taste receptors. These receptors could help to monitor the composition of blood, CSF and interstitial fluid, and, through transporters upon ligand-binding, to control alterations in brain fluid composition [16].

### Cerebrospinal fluid production and transport systems in the B-CSF barrier

A classic and widely-accepted hypothesis claims that the primary function of CP epithelial cells is secretion of cerebrospinal fluid (CSF) into the brain ventricles. Much literature has been produced pointing to the likelihood that indeed the CP may produce more than 50% of total CSF [17–23]. The remaining CSF is derived from the interstitial fluid of the brain which is produced by the BBB and the ependymal cells lining the ventricles [18, 24–26]. The total volume of CSF in humans is about 140 ml, and every day about 600 ml CSF is produced [27].

The formation of CSF in CP depends mainly on Na^+^, K^+^, Cl^−^, HCO^3−^ and H_2_O transport. The osmotic gradient created by ion movement drives the secretion of water [28–31]. A number of transporters were found on the apical membrane of the CP epithelial cells, including K^+^ channels (Kv1.3; Kv1.1 and Kir7.1), Na^+^-HCO^3−^ cotransporter (NBCe2), Na^+^-K^+^-2Cl^−^ cotransporter (NKCC1), the K^+^-Cl^−^ cotransporter KCC4, Na^+^/H^+^ exchanger (NHE1), Cl^−^ channels (VRAC and Clir), Na^+^-K^+^-ATPase and aquaporin (AQP1). Interestingly, only a few transporters were found on the basolateral membrane of the CP epithelium including the K^+^-Cl^−^ cotransporter KCC3, Na^+^-HCO^3−^ cotransporters (NBCn1 and NCBE/NBCn2), Cl^−^/HCO^3−^ exchanger (AE2), glucose transporter-1 (GLUT1) and AQP1 [18, 32–36].

NKCC1 that is expressed in the apical membrane of CP may contribute as much as half of the CSF production, via ion-mediated cotransport of water [37, 38].

Transcellular water transport is supported by a family of AQPs. AQP1 is expressed in the basolateral membrane of the CP epithelial cells but at much lower levels than those in the apical membrane [39–41]. Based on experimental findings, it seems that AQP1 contributes to the production of 20–25% of the CSF [42]. AQP4 is expressed in the cytoplasm but not in the apical or basolateral membrane of CP epithelial cells. Diffuse cytoplasmic distribution of AQP4 and AQP5 suggests their possible expression in the membranes of intracellular organelles. Evidence of AQP7 in the apical membrane of the CP cells suggests a possible cooperation of AQP7 with AQP1 in CSF secretion [38, 43–45]. It was also suggested that GLUT1 may increase the permeability of the basal membrane of CP epithelial cells to water [19, 38].

CSF secretion is influenced by the action of the autonomic nervous system. The capillaries and cuboidal epithelial cells are innervated by adrenergic nerve fibers arising from the superior cervical ganglia [46, 47]. In addition, CP capillaries and cuboidal epithelial cells are also innervated by parasympathetic nerve fibers probably related to the glossopharyngeal and vagus nerves [48, 49]. Parasympathetic stimulation reduces Na^+^-K^+^ ATPase activity mediated by nitric oxide formation, and results in reduced CSF secretion [50]. Moreover, serotoninergic and peptidergic fibers in the CP also possibly influence CSF production [48, 51, 52].

Specific transport mechanisms employing a variety of transmembrane proteins that enable selective passage of molecules form important components of the B-CSF barrier. They are crucial for the exchange of metabolites and xenobiotics between blood and CSF. These transporters mainly belong to the ATP-binding cassette (ABC) transporter and solute carrier (SLC) super-families [21]. Two main types of ATP-biding cassette (ABC) transporters are expressed in the CP; the multidrug resistance-related proteins (MRPs) and multidrug transporters P-glycoprotein (PgP) [53]. The MRPs are selectively expressed on the apical or basolateral side of the epithelial cells, and move metabolic waste products and harmful molecules out of the CNS. The MRP1 protein transporter contributes to the protection of the B-CSF barrier against heavy metal ions, toxins, and various xenobiotics [54–56]. On the other hand, PgP is localized on the apical side of CP epithelial cells, where if anything, might aid the entry of harmful molecules into the CSF as was described for Taxol and ^99m^Tc-sestamibi [57, 58]. The CP PgPs are shown to have a different pumping function than those in the BBB [4, 59–64]. SLC transporters also play significant roles in the neuroprotective machinery of the B-CSF barrier. The organic cation transporter SLC29A4 removes serotonin, dopamine and histamine from the CSF, and thus may be involved in the termination of neurotransmitter signaling [21].

In addition, it was shown that a wide range of transporter proteins expressed in the CP are potentially involved in the maintenance of the metabolite- and xenobiotic- balance in the CSF and CNS. Organic anion transporting polypeptide (Oatp), together with PgP, is known to keep certain drugs in the CSF at a low subtherapeutic level. Low density lipophilic receptor-related proteins (LRP) localised to the apical side of the CP epithelial cells are involved in the clearance of peptides and peptide fragments such as amyloid precursor protein from the CSF [65]. MRP4 and five, large neutral amino acid transporter LAT1/slc7a5, Menke’s and Wilson’s metal transporters, divalent metal transporter (DMT)-1, equilibrative nucleotide transporters (Ent)-1, peptide transporters (Pept)-2, and proton-coupled oligopeptide transporter slc15a2/Pept2 are shown to be highly expressed in the CP epithelial cells [20, 66–70].

### Intercellular junctions in the CP

The B-CSF barrier is formed from the different types of cell junctions between the epithelial cells of the CP. The regulation of B-CSF barrier permeability and integrity is the fundamental role of apical tight junctions (TJs) of CP epithelial cells—they are responsible for regulating paracellular diffusion of water-soluble molecules through this barrier [71, 72]. Moreover, TJs maintain electrical resistance across the epithelial layer of the CP [72, 73]. The TJs are composed of proteins associated with the inner and outer leaflets of the cell membrane [74], whereas occludin and claudins are the main transmembrane molecules mediating epithelial contact [75]. The first integral protein of TJ to be described in CP was occludin [76, 77]. There is evidence that occludin seals the TJs, as decreased occludin levels lead to disruption of TJ permeability [78]. The group of TJ proteins called claudins are divided into several subtypes and CP epithelial cells express claudin 1–6, 9–12, 19 and 22 [77, 79, 80]. They are integral proteins responsible for limiting the throughput of TJ [81, 82].

**Fig. 2:**
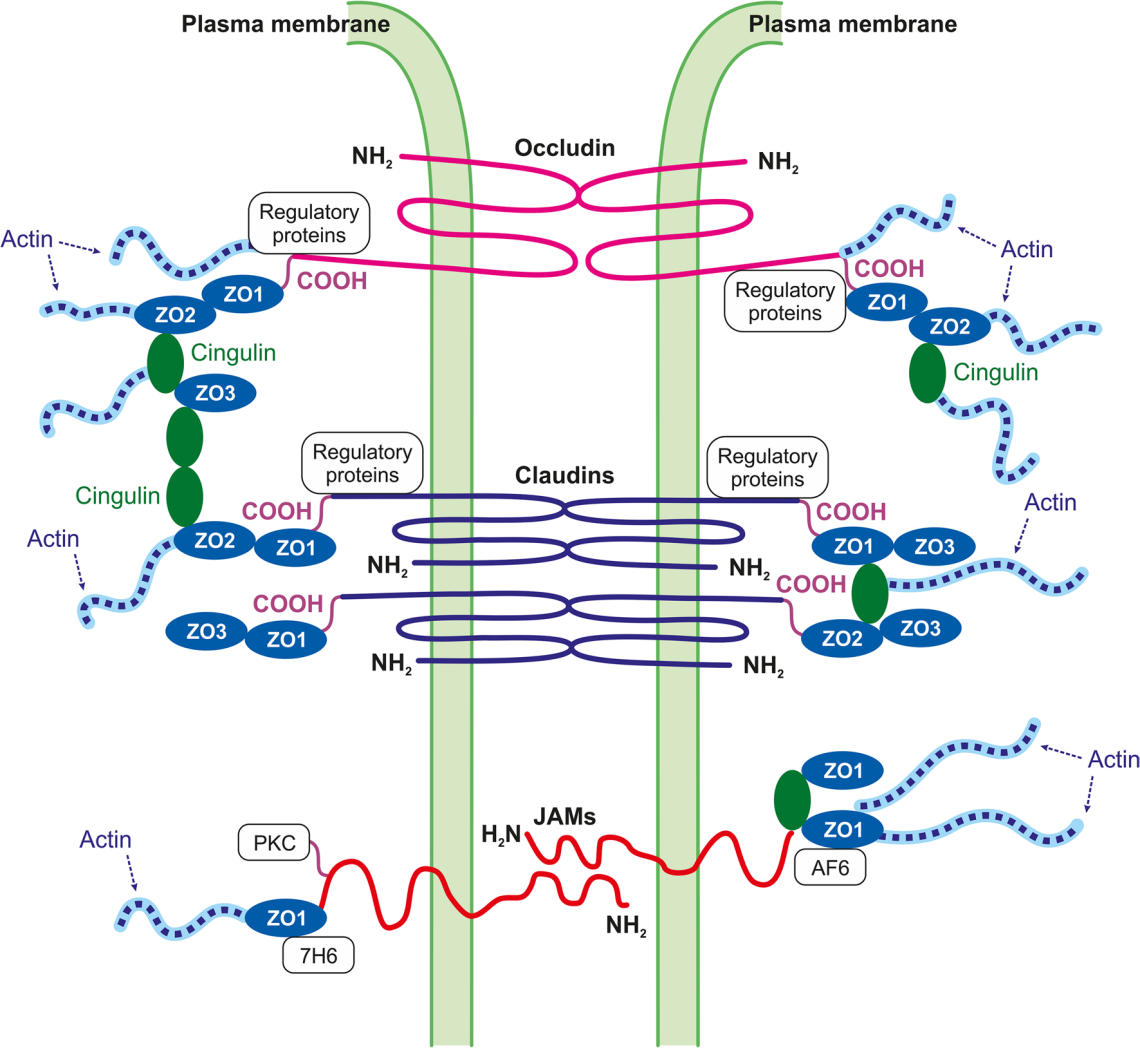
Schematic illustration of the molecular interactions of TJ proteins in CP epithelial cells. TJ proteins and their complex interactions with other proteins form the connections between adjacent epithelial cells in the B-CSF barrier. Claudin, occludins, and JAMs are linked to actin filaments through ZO proteins. Cingulin, another cytoplasmic protein, is recruited to TJ through ZO. The interaction between TJ proteins and the cytoplasmic protein network is crucial for maintaining B-CSF barrier integrity (adapted from [72])

Zonulin (ZO) is a sub-membranous component TJ protein in mammalian epithelia including in the cuboidal CP epithelial cells [83, 84]. Three ZO proteins, ZO-1 [85, 86], ZO-2 [87, 88] and ZO-3 [89, 90] bind occludin and claudin to actin filaments [91, 92]. The ZO proteins are members of the membrane associated guanylate kinases (MAGUK) family that have three domains: protein-binding modules (PDZ domains), a domain that mediates critical protein–protein interactions (SH3 domain) and a guanylate kinase (GuK) domain ([91, 93, 94]; Fig. 2).

Adherent junctions (AJs) are localized on the basolateral membrane of CP epithelial cells. They regulate cell contact and determine the polarity of cells in the brain barriers. AJs are formed by the homophilic interaction of transmembrane proteins in a calcium-dependent manner—E-cadherin linked to the actin cytoskeleton via interaction with other proteins like p120, β-catenin, α-catenin, and afadin-6. These AJ proteins are essential prerequisites for the development of TJs [72, 84, 95]. Lobas et al., demonstrated that CP epithelial and ependymal cells express highly the 22-member γ-protocadherin family of cell adhesion molecules that play a significant role in the production, dynamics, and composition of CSF [96].

The regulation of the B-CSF barrier is also affected by gap junctions (GJs), composed of intercellular protein channels between cells. These channels are formed by two hemichannels that are composed of six connexin proteins [97]. GJs mediate intercellular communication, exchange of metabolites, electrolytes, and second messengers between adjacent epithelial cells [98–100].

## B-CSF barrier in infectious diseases

### Systemic inflammation

Systemic administration of the bacterial endotoxin lipopolysaccharide (LPS)—a component of the outer membrane of Gram-negative bacteria—is the most commonly used model of systemic inflammation.

Monitoring gene expression in the CP after systemic LPS administration showed that the up-regulated genes are mainly involved in immune modulation and extracellular matrix remodeling, whereas those down-regulated play a role in maintaining barrier function [101]. The acute phase of the CP reaction leads to increased expression of IL-1β and TNF-α 1 h after LPS application while prostaglandin D2 synthase (LPTGDS) that mediates both pro- and anti-inflammatory actions was detected between 6 and 24 h after LPS administration [102]. The protein lipocalin 2 (LCN2) was found exclusively in CP epithelial cells, peaking after 12 h and returning to basal levels 72 h after LPS administration [103, 104]. LCN2 binds a negatively charged ferric siderophore and exerts a bacteriostatic effect by sequestering iron [105, 106]. It seems that limiting iron availability to microorganisms is an important strategy to prevent translocation of microorganisms into the CNS. This suggestion is supported by overexpression of iron-related genes like hepcidin (Hamp), ceruloplasmin, ferritin heavy chain 1 (Fth1), and other genes encoding for upstream regulators of Hamp expression like Stat3, Smad4, Tfr2, and IL-6 in the CP epithelial cells after LPS administration [107].

It was found that LPS with LPS-binding protein (LBP) binds to CD14 on the cell membrane. This complex transfers LPS to myeloid differentiation factor 2 (MD-2) and toll-like receptor-4 (TLR4), leading to LPS internalization and activation of NF-κB and the mitogen-activated protein kinase (MAPK) pathway [108, 109]. Studies showed that systemic injection of LPS resulted in upregulation of TLR1, TLR3, TLR4, CD14, COX-2, IκBα in the CP cells which may lead to decreased expression of occludin [110–116]. In addition, there is evidence that activation of NF-κB upregulates matrix metalloproteinase-9 (MMP-9) production and induces claudin-5 degradation leading to alteration of the B-CSF barrier [117]. The expression of both MMP-8 and MMP-9 is upregulated 4 h after LPS application. Nevertheless, MMP-8 probably acts via collagen I cleavage, which affects the composition of the basal lamina of the CP and influences epithelial cell morphology and barrier integrity [118].

It seems that expression of NF-κB, along with upregulation of TNFα may induce alteration of B-CSF barrier. It was found that elevated expression of TNFα and the subsequent response through NF-κB following LPS treatment is time- and dose-dependent [119, 120]. CP responds to systemic administration of LPS by increasing mRNA expression of TNFα receptor types I and II as well as IL-1β and its two receptors, IL-1 receptor type I and II, as well as IL-6 and its signal transducing component [121].

Systemic inflammation leads to recruitment of inflammatory cells in the CP. It has been suggested that monocyte chemoattractant protein-1 (MCP-1) known as CCL2 is primarily responsible for the accumulation of leukocytes in the CP after systemic LPS administration. Increased expression of MCP-1/CCL2 was found in the acute phase following even a single systemic LPS application [122]. Nevertheless, repeated systemic inflammatory stimuli also lead to increased expression of molecules required principally for trafficking of blood-borne immune cells into the CP. The expression of intercellular adhesion molecule 1 (ICAM1), glycosylation dependent cell adhesion molecule 1 (GlyCAM1), mucosal addressin cell adhesion molecule 1 (MAdCAM1), junction adhesion molecule 2 (Jam2), selectin P ligand (Selpl), the chemokines CXCL1, CCL7, CCL2 and interleukins such as IL-16 was increased after repeated systemic LPS administration over a period of 3 months [123, 124].

Another study using CP primary cell cultures stimulated with LPS showed increased secretion of extracellular vesicles (EVs) containing inflammatory miRNAs. Analysis of EVs revealed LPS-dependent miR-9, miR-146a, and miR-155 up-regulation and miR-1a down-regulation. On the other hand, miRNA expression showed miR-1a/-9 down-regulation and miR-146a/-155 up-regulation in LPS-stimulated primary CP epithelial cells [125].

### CP infection and pathogen invasion

**Fig. 3:**
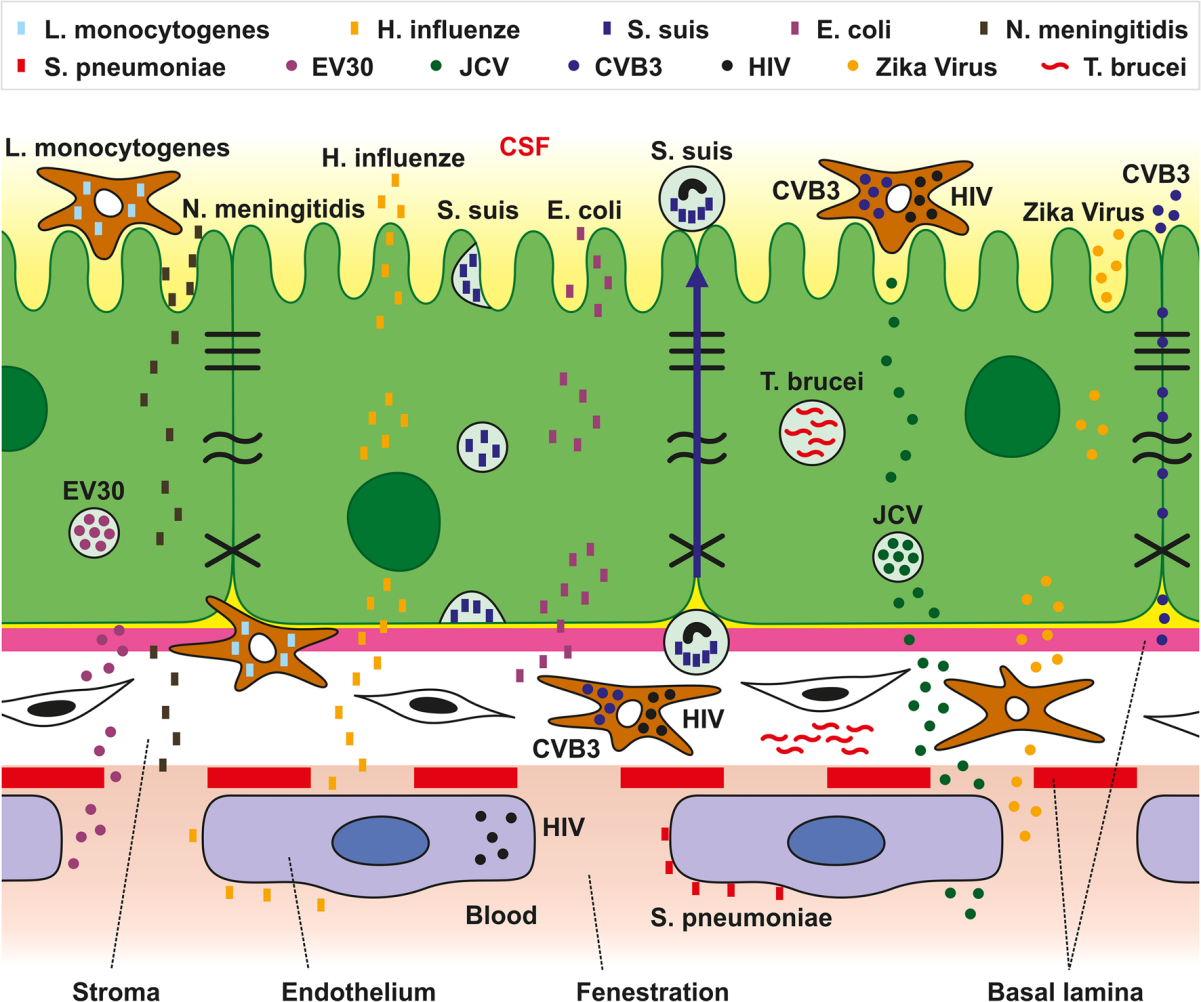
Schematic illustration showing the various invasion strategies of different pathogens (bacteria, viruses and parasites) through the B-CSF barrier into the CNS. *Streptococus suis* (*S. suis)* can cross the B-CSF barrier within endocytic vacuoles and there is some evidence supporting a “Trojan horse” mechanism using polymorphonuclear leukocytes. *Streptococus pneumoniae* (*S. pneumoniae)* interacts with the endothelium of the CP. *Listeria monocytogenes (L. monocytogenes)* has been observed invading the CNS using a “Trojan horse” mechanism inside mononuclear cells. *Escherichia coli (E. coli)* and *Haemophilus influenzae (H. influenzae)* can migrate through CP epithelial cells. *Neisseria meningitidis (N. meningitidis)* crosses the B-CSF barrier and forms colonies at the apical side of CP epithelial cells. *Polyomavirus JC (JCV)* probably forms a reservoir in CP epithelial cells. *HIV* has been described in endothelial and stromal cells as well as in epiplexus monocytes. *Echovirus 30 (EV30)* may invade and replicate in CP epithelial cells. *Coxsackievirus B3 (CVB3)* crosses the B-CSF barrier using myeloid cells as well as via a paracellular route through the TJs. *Trypanosoma brucei (T. brucei)* was found in the perivascular region of the CP and also in CP epithelial cells

Infections of the CNS can be induced by various pathogens such as viruses, bacteria, fungi, and parasites. These pathogens may access the CNS via different routes such as migration into the cells, through a loosened TJ leading to paracellular transport or they can exploit infected phagocytic host cells—the so-called “Trojan horse” strategy (Fig. 3). Pathogen invasion through the B-CSF barrier is followed by chemokine secretion and results in immune cell trafficking through the CP epithelium and the consequent development of CNS inflammation (Table 1; [126, 127].

**Table 1:** Table summarizing CP changes in inflammatory diseases

Category	Disorder	Choroidal epithelial cells	Junctions	Immune cells	Transporters	Others
Inflammation and infection	Systemic inflammation	↑ COX-2 and IκBα expression [113, 115]	↓ Occludin mRNA expression [116]			↑ CCL2 expression [122]
		↑ TLR1, TLR3, TLR4, CD14 expression ↓ Occludin expression [110–112, 114, 116]				↑ Expression of ICAM-1, GlyCAM-1, MAdCAM-1, Jam2, Selpl, chemokines CXCL1, CCL7, CCL2, IL-16 [123]
		↑ TNFα expression [119, 120]				Collagen I cleavage [118]
		↑ Il-1β, TNFα, LPTGDS expression [102]				↑ mRNA expression of TNFRI, TNFRII, IL1β, IL1 receptor type I, type II, Il6 and its signal transducing component – Il6 signal transducer [121]
		↑ LCN2 expression [103, 104]				
		↑ Expression of Hamp, Cp, Fth1, Stat3, Smad4, Tfr2, Il6 genes [107]				
		↑ MMP-8 expression [118]				
		↑ EVs secretion [125]				
	Bacterial infections	(*Streptococcus suis*) fibrinous exudate, disruption of epithelial “brush border” [134]	(*Streptococcus suis*) Disruption of normal pattern of the TJ—Occludin, ZO-1, claudin-1 [128]	↓ (*Streptococcus suis*) Number of epiplexus cells [134]	(*Haemophilus influenzae*) Paracellular invasion into CSF [145]	↑ (*Streptococcus pneumoniae*) NF-κB, PAF, laminin receptor expression in CP endothelial cells [136]
		↓ (*Streptococcus pneumoniae*) TNFα -mRNA expression [138]	↓ (*Streptococcus suis*) Claudin-2 expression [131]		(*Listeria monocytogenes*) Using the “Trojan horse” mechanism by occupying mononuclear cells in the CP [139]	
		(*Streptococcus suis*) Apoptosis and necrosis of CP epithelial cells [132]			(*Streptococcus suis*) Transcellular migration in endocytic vacuoles [133]	
		(*Listeria monocytogenes*) Interaction of internalin A and B with E-cadherin on CP epithelial cells [140]			(*Streptococcus suis*) Transepithelial migration of polymorphonuclear leukocytes [135]	
		↑ (*Streptococcus suis*) ICAM-1, VCAM-1, MMP-3, NFκB, MAPK, TNFα, Il-1β, IL-6, IL-8, LIF, ARG1, ARG2, NOS2, indoleamine 2,3-dioxygenase expression [129–131]			(*Neisseria meningitidis*) Migration through CP epithelial cells [148]	
		(*Streptococcus pneumoniae*) Antibacterial effect of TLR2 [136]			(*Escherichia coli*) Invasion through CP epithelial cells [144]	
		↑ (*Neisseria meningitidis*) IL6, CCL20, CXCL1-3, Nfkbiz, GM-CSF expression [150]			(*Haemophilus influenzae*) Transcellular migration from the basolateral side of CP epithelial cells [146]	
		(*Listeria monocytogenes*) Activation of MAPKs, Erk1/2 and p38 [142]				
	Viral infections	(Echovirus 30) Invasion and replication in CP epithelial cells [161]		(HIV) MHC II + dendritic cells in the CP as reservoir of HIV [160]	(Zika virus) Entry to the brain through CP epithelial cells [152]	(HIV) Endothelial cells in the CP as a reservoir of HIV [159]
		↑ (Echovirus 30) CXCL3, CXCL10, CXCL11, CCL20, IL8, IL7, M-CSF expression [162]		(HIV) Monocytes-like cells in the CP as a reservoir of HIV [156]		
		(Polyomavirus JC) CP cells as a reservoir [153]		(Coxsackievirus B3) Diapedesis of infected myeloid cells expressing high level of Ki67 and pERK1/2 through the TJ of the CP [163]		
	Fungal infections					(*Cryptococcuis neoformans*) CP plexitis in HIV patients [170, 172]
	Parasitic infections	(*Trypanosoma brucei*) Present in perivascular region of CP and in CP epithelial cells [174]		(*Leishmania chagasi*) Infiltration of inflammatory cells with lymphoplasmatic morphology around the blood vessels and diffusely in CP stroma [176]		(*Leishmania chagasi*) Deposits of perivascular hyaline substances [176]
		(*Trypanosoma evansi*) Edema and rupture of the CP epithelial cell layer [175]		(*Trypanosoma evansi*) Infiltration of CP by inflammatory cells [175]		↑ (*Leishmania infantum*) TLR2 and TLR9 expression, no changes in TLR4 expression [177]
				(*Leishmania infantum*) Inflammatory infiltration via CD3+ T lymphocytes [176]		(*Leishmania infantum*) Accumulation of perivascular hyaline substances consistent with IgG and parasite DNA [176]

### Bacterial infections

It seems that CP reacts to infection by *Streptococcus suis* (a gram-positive bacterium) by different mechanisms. *Streptococcus suis* infection induces disruption of epithelial integrity through the rearrangement of the TJ proteins occludin, ZO-1 and claudin-1. They then start to appear throughout the lateral membrane of the cells and within the cytoplasm of CP epithelial cells. These changes include shift of the perijunctional actin belt to the basal segment of the lateral membranes, flattening of cells, and “bundles” of actin stress fibers appearing in the basolateral cell compartment indicating restructuring of polarized F-actin. Occludin was not localized at the perijunctional actin belt, whereas a subset of actin fibers remained colocalized with ZO-1 [128]. In CP epithelial cells, the gene coding for claudin-2 was strongly down-regulated by *Streptococcus suis*. On the other hand, some genes in the CP epithelial cells were highly up-regulated including genes encoding adhesion molecules like ICAM-1, vascular cell adhesion molecule-1 (VCAM-1), MMP-3, the NF-κb and MAPK inflammatory cascades, inflammatory molecules like TNFα, IL1-ß, IL-6, IL-8 as well as the anti-inflammatory leukemia inhibitory factor (LIF). Moreover, infection by *Streptococcus suis* induces up-regulation of genes encoding proteins that participate in arginine-metabolism (ARG1, ARG2, NOS2). In addition, up-regulation of indoleamine 2,3-dioxygenase that contributes to antimicrobial defense in the CP by depleting tryptophan was observed [129–131]. Moreover, *Streptococcus suis* induces cell death in CP epithelial cells by apoptosis and necrosis. Intensified cell death in the CP could lead to alteration in the B-CSF barrier function and facilitate penetration of bacteria and leukocytes into the CSF of the ventricular system reaching up to the brain parenchyma [132]. Another mechanism by which *Streptococcus suis* may enter the CSF involves invasion from the basolateral side of CP epithelial cells, transport within endocytic vacuoles to the apical side and exocytosis on the apical membrane. It has been shown that downregulation of PI3K effector molecules including the protein kinase Akt and members of the protein kinase C family participate in this transcellular migration of pathogens like *Streptococcus suis* [133].

Transcellular migration is supported by the detection of *Streptococcus suis* within the CP epithelial cells and in the ventricular exudate, but not on the surface of CP epithelial cells. In addition, morphological changes including disruption of the epithelial “brush border” and decreased numbers of epiplexus KCs was also shown [134]. Moreover, transepithelial migration of polymorphonuclear leukocytes through the B-CSF barrier was reported following *Streptococcus suis* infection and TNFα stimulation supporting a possible “Trojan horse” mechanism [135].

*Streptococcus pneumoniae*, a gram-positive bacterium, adheres to blood vessels including those of the CP in conditions of bacteremia. Interestingly, no *Streptococcus pneumoniae* was detected in the early stage of infection, while at 8 and 14 h after infection the bacteria were found associated with CP vessels. This may be caused by increased basal levels of platelet-activating factor (PAF), laminin receptors or the NF-κB cascade during infection [136]. On the other hand, adhesion and accumulation of *Streptococcus pneumoniae* on CP epithelial cells is reduced by TLR2 that may accelerate granulocyte recruitment and bacterial clearance [137]. Rapid bacterial uptake could be the reason why there were no TNFα-mRNA positive CP epithelial cells in a model of pneumococcal meningitis [138].

*Listeria monocytogenes* is a facultatively gram-positive intracellular bacterium. It was proved that this species uses the “Trojan horse” mechanism by hijacking mononuclear cells [139]. *Listeria monocytogenes* makes two surface proteins, internalin A and B. Interestingly, the interaction of these surface proteins with their human receptor E-cadherin, seems to be involved in entry of *Listeria monocytogenes* also into cultured human epithelial cells and deletion of these proteins leads to a reduction in *Listeria* invasion [140, 141]. Activation of MAPKs Erk1/2 and p38 signaling preferentially from the basolateral side of the CP epithelium has been described in an *in* *vitro* model of the B-CSF barrier based on human CP papilloma cells following exposure to *Listeria monocytogenes*. Concurrent inhibition of MAPK signaling pathways potentially reduces infection [142].

*Escherichia coli* is a common gram-negative bacterium. Studies showed that *Escherichia coli* expresses fimbriae recognizing sialyl galactosides (S-fimbriae) that adhere on the endothelia and the epithelial lining of the CP [143]. A recent study has shown that bacterial invasion into CP epithelial cells occurs exclusively on the basolateral cell side. Several virulence factors like outer membrane protein A (ompA), invasion protein ibeA and neuDB facilitate the infection, and type 1 fimbrin d-mannose specific adhesin (fimH) accelerates the invasion through CP epithelial cells [144].

*Haemophilus influenzae* is a gram-negative, facultatively anaerobic bacterium that has the

ability to adhere through S-fimbriae to the luminal surfaces of the vascular endothelium and of the epithelium lining the CP and may take advantage of this ability to enter the CSF [145]. In a recent study it has been proved that *Haemophilus influenzae* can invade the CP epithelial cells from the basolateral side and this capacity is attenuated by the presence of virulence factors like fimbriae and capsule. Down-regulation of the capsule seems to be favorable for cellular entry [146].

The mechanisms of CNS invasion of *Neisseria meningitidis* (gram-negative bacteria) is not yet clear [147]. It has been suggested that *Neisseria meningitidis* invades CP epithelial cells from the basolateral side, and after migration through the cells, forms colonies on the apical side of the CP cells [148]. On the other hand, careful examination of CP sections did not reveal any bacteria inside or between CP epithelial cells. However, most CP vessels contained bacteria interacting with endothelial cells. The high level of tip-located adhesin proteins PilC1 and PilC2 of these bacteria implies a role in enhanced adhesiveness to the CP [149]. It was also found that *Neisseria meningitidis* can induce increased expression of IL-6, CCL20, CXCL1-3 and *Nfkbiz* (encoding the nuclear IκB protein IκBζ), as well as granulocyte colony stimulating factor (G-CSF) and granulocyte–macrophage colony-stimulating factor (GM-CSF) in CP epithelial cells. Activation of the IκBζ pathway probably occurs via the TLR2/TLR6 signaling pathway [150].

### Viral infections

Viral meningitis occurs more frequently than bacterial meningitis and could be caused by several types of viruses [151]. Various neurotrophic viruses have recently come under investigation in relation to the CP. It has been found that the Zika virus enters the brain from the blood through epithelial cells in the CP without active replication [152]. Polyomavirus JC (JCV) may first reach CP cells through the bloodstream and then be released into the CSF. JCV could use CP cells as reservoirs thus facilitating progressive multifocal leukoencephalopathy or other JCV-related encephalopathies [153].

The CNS is also attacked by Human Immunodeficiency Virus 1 (HIV-1) systemic infection [154]. Previous studies have demonstrated that the CP serves as a long-term reservoir for HIV despite antiviral therapy [155, 156]. HIV positive cells were found in CP stroma, capillary endothelium as well as in the epiplexus position. Based on this finding it was suggested that HIV encephalitis may develop by allowing entry of HIV infected monocytes from blood to the CP and CSF [156–158]. In addition, microvascular endothelial cells may be another reservoir for the virus and may also facilitate virus entry across the B-CSF barrier [159]. It has been suggested that dendritic cells in the CP are strongly immunoreactive for the class II MHC antigen HLA-DR, and could play a major role in the pathogenesis of HIV encephalitis by serving as a reservoir of HIV in the CNS [160].

Echovirus 30 (enteric cytopathic human orphan virus; EV30), is one of the most common serotypes of enteroviruses causing meningitis especially in children, and has been shown to invade and replicate in CP epithelial cells. EV30 can infect CP epithelial cells from both the apical and basolateral side [161]. Infection with EV30 results in the upregulation of the chemokines CXCL-3, -10, -11, -20 as well as IL-8 and macrophage colony-stimulating factor (M-CSF). During EV30 infection, there is predominantly a polar direction of cytokine/chemokine secretion in the basolateral side of the CP, with only IL-7 being secreted on the apical side to the CSF [162].

Coxsackievirus B3 (CVB3) can establish a persistent infection and cross the B-CSF barrier using specific myeloid cells, while CP epithelial cells remain unaffected. The migration of infected myeloid cells may lead to a productive mechanism of virus propagation within the CNS. The myeloid cells are highly susceptible to infection once they cross the CP epithelial TJ proteins. Moreover, numerous infected myeloid cells express high levels of Ki67 and pERK1/2 suggesting their proliferation. Binding of TJ-located coxsackie and adenovirus receptor by CVB3 virions assists virus spread into the CNS [163].

Severe acute respiratory syndrome coronavirus 2 (SARS-CoV-2), causing the coronavirus disease 2019 (COVID-19), was recently described by the World Health Organization (WHO) [164]. It was found that SARS-CoV-2 can also infect the CNS [165]. One of the possible pathways for the SARS-CoV-2 to enter the CNS may be via hematogenous spread and crossing the BBB or B-CSF barrier [166].

### Fungal infections

*Cryptococcus neoformans* is the most common fungus opportunistically infecting the CNS in HIV positive patients. Both direct transcellular migration and “Trojan horse” models of penetration to the CNS has been described. It appears that invasion into the brain occurs via the BBB from among cortical capillaries but not through the CP [167–169]. However, co-occurrence of CP plexitis and ependymitis in HIV patients reflecting immune activation in the CP has been shown via magnetic resonance imaging (MRI) [170, 171]. Finding CP plexitis in the MRI in immunocompromised patients may indicate a diagnosis of CNS cryptococcosis [172].

### Parasitic infections

The BBB is major entry site for parasite invasion into the brain [173]. However, there is evidence indicating that *Trypanosoma brucei* enters the brain via the CP and CSF. This parasite was detected in the perivascular region of the CP and in CP epithelial cells [174]. A histopathological study of brain and the CP after *Trypanosoma evansi* infection showed extensive edema, rupture of the CP epithelial cell layer and infiltration of the CP by inflammatory cells [175].

Morphological alteration of the CP was also described in visceral leishmaniosis caused by the parasitic protozoan *Leishmania chagasi*. These alterations include inflammatory infiltration of the CP by CD3+ T-lymphocytes and accumulation of perivascular hyaline consistent with IgG and parasite DNA deposition [176]. Further, *Leishmania infantum* induces significantly increased expression of TLR2 and TLR9 in the CP [177].

## The CP and B-CSF barrier in stroke

Generally, studies describing changes to the CP after hemorrhagic or ischemic injury are focused on alteration in CSF production and disruption of B-CSF barrier (Table 2).

**Table 2:** Table summarizing changes of the CP in stroke and nervous system trauma

Category	Disorder	Choroidal epithelial cells	Immune cells	Transporters	Others
Stroke	Hemorrhagic	↑ NF-κB expression [299]	↑ Number of macrophages in the epiplexus position [184]	↑ Na^+^/K^+^/2Cl^−^ co-transporter 1 regulation [190]	↓ CSF secretion [198]
		↑ mRNA for NF-κB, MCP-1, IL1R1, IL-8, IL-6, FAS, TNF-α expression [183]	↑ Iba-1+ and CD68+ epiplexus cells [186]		↑ Number of cytoplasmic water vesicles [191–193]
		↑ HO-1 expression [183]		↑ AQP1 expression [194]	
		Activation of TLR4 [182]			
	Ischemic	Apoptosis or necrosis according to the severity of ischemic injury [201]	↑ ED1+ number, CR3 receptors, expression of MHC I and MHC II antigens [217]	↑ Calcium transfer to CSF [210]	Alteration in B-CSF barrier after middle cerebral artery occlusion [207]
		↑ Apoptotic cells characterized by nuclear DNA fragmentation after middle cerebral artery occlusion [201]	↑ iNOS expression in epiplexus cells, Number of OX-42+ , ED1+ , OX-18+ , OX-6+ cells in epiplexus position [217]		↑ Permeability of B-CSF barrier for inulin after bilateral carotid occlusion [205]
		↑ Expression of TGFβ1, brain-derived neurotrophic factor and other growth factors [216]	↑ MMP-9 expression in infiltrating macrophages [214]		↑ VEGF and eNOS expression [217, 218]
		Plasma membrane and organelle damage; clumping of nuclear chromatin after forebrain ischemia [205, 206]	↑ Number of macrophages [220]		
		↑ CP epithelial cell proliferation Expression of NeuN and GFAP after middle cerebral artery occlusion [215]	CP as invasion route for T cells into the ischemic brain [219]		
		Edema, apoptosis of CP epithelial cells after middle cerebral artery occlusion [212]			
		↑ eNOS, iNOS, nNOS expression and accumulation of glycogen [217]			
		↑ Secretion of growth factors including GDNF, BDNF, NGF [222–224]			
		↑ MMP-9 level, vacuolization Indistinct epithelial membranes and varying degrees of pyknosis after middle cerebral artery occlusion [214]			
		Desquamation of CP epithelial cells [199]			
				↓ AQP1 expression up to 24 h ↑ AQP1expression between 24–48 h after global cerebral ischemia [209]	
		↑ VCAM-1, MAdCAM-1, C3CL1, Nt5e expression [220]			
Traumatic	TBI	↑ Intercellular spaces between CP epithelial cells [225]	↑ Macrophage number in epiplexus position after non-penetrative injury [227]		Changes in CSF composition [300]
		CP apical membrane ruptured [227]			
		Uptake of serum albumin by CP epithelial cells [226]			
	Spinal cord injury		↑ Trafficking of M2 macrophages to the injured spinal cord supported by Vcam-1-VLA4 and epithelial CD73 [231]		
	Peripheral nerve injury		↑ Number of ED1+ and ED2+ macrophages in the CP [232]		

### Hemorrhagic stroke

The CP responds to hemorrhagic stroke including intraventricular hemorrhage (IVH) and subarachnoid hemorrhage by an inflammatory reaction. It is known that degradation of blood components like heme, fibrinogen and intracellular structures creates damage-associated molecular patterns (DAMPs) that trigger inflammatory signaling cascades [178, 179]. One possible inflammatory reaction that is triggered by DAMPs is activation of TLRs that play a key role in the innate immune reaction [180, 181]. In a model of IVH, DAMPs led to activation of TLR4 in CP epithelial cells resulting in hypersecretion of CSF. It seems that this hypersecretion is dependent on TLR4/NFκB signaling, activation of Ste20-related proline alanine-rich kinase (SPAK) and Na^+^/K^+^/2Cl^−^ co-transporter 1 involved in CSF secretion [182]. It appears that upregulation of the transcription factor NF-κB and chemoattractant molecules like CCL2, IL-1β, IL-6, IL-8 and TNFα, in CP epithelial cells support monocyte trafficking into the CP [183].

It has been found that subarachnoid hemorrhage (SAH) increases the number of macrophages in the epiplexus position of the CP, but the type of macrophages was not specified [184]. We described that SAH induces an immune reaction in the CP resulting in an increase in the number of M1 (ED1+ , CCR7+) and M2 (ED2+ , CD206+) macrophages as well as MHC-II+ antigen presenting cells in the epiplexus position. Moreover, we also found that increased intracranial pressure due to artificial CSF application induced both an immune reaction and increased proliferation of epiplexus cells in the CP. These findings indicate that increased intracranial pressure, and not just blood, contributes to cellular changes in the CP following SAH [185]. A recent study showed that SAH induces an increased number of ionized calcium-binding adapter molecule 1 immunopositive (Iba-1+) cells and activated (CD68+) epiplexus cells [186]. Accumulation of immune cells in the CP after SAH or IVH triggers inflammation, alteration of TJ proteins and hypersecretion of CSF. This pathophysiological cascade may contribute to the development of post-hemorrhagic hydrocephalus—one of the main complications after hemorrhagic stroke [182, 187, 188]. It was believed that development of posthemorrhagic hydrocephalus is due to obstruction of CSF circulation, inhibition of CSF drainage in the arachnoid villi or meningeal fibrosis [189]. In contrast, other results suggest that CSF hypersecretion can also potentiate post-hemorrhagic hydrocephalus after SAH or IVH. A possible mechanism may be via upregulation of Na^+^/K^+^/2Cl^−^ co-transporter 1 in the cuboidal epithelial cells [190]. Hypersecretion of CSF may also be induced by stimulation of glossopharyngeal and vagus nerve endings that innervate the CP epithelium and arteries. An increase of cytoplasmic water vesicles in the acute phase of SAH is probably due to irritation of both nerves [191–193]. Moreover, a recent study suggests that AQP1 may also contribute to hypersecretion of CSF and post-hemorrhagic hydrocephalus. A significantly higher expression of AQP1 was found in the apical and basolateral membrane of CP epithelial cells from 1 to 14 days peaking at day 3 following the induction of SAH [194].

CP epithelial cells and epiplexus macrophages are in direct contact with blood elements and their degradation products after SAH or IVH. Heme-oxygenase (HO) and biliverdin reductase (BVR) are the main enzymes involved in hemoglobin degradation [195]. HO-1, an isoform of HO, is the main enzyme responsible for clearance of blood from the subarachnoid space after SAH [196]. HO-1 increases in the CP epithelial cells in response to the presence of blood in the cerebrospinal fluid after IVH [183]. The presence of BVR has been described in brain tissue in co-expression with HO isoforms [197]. However, the dynamics of BVR expression in the CP after SAH or IVH has not been reported.

Vasospasm of the CP arteries was induced 20 days after SAH followed by ischemic injury, apoptosis of CP cells and decreased secretion of CSF [198]. It seems that degeneration of CP cells increases in severity depending on the volume of blood in the subarachnoid space [199]. Nevertheless, hydrocephalus may also occur in the absence of CSF hypersecretion probably due to desquamation of the ependymal and CP epithelial cells leading to reduction of cerebral aqueduct volume [200].

### Ischemic stroke

CP reacts to ischemia by functional impairment, apoptosis and/or necrosis of CP epithelial cells depending on the severity of the underlying ischemia [201]. Effects of hypoxia/ischemia on the CP were described using two cerebral ischemia experimental models characterized as global and focal. Global cerebral ischemia reduces cerebral blood flow throughout most of the brain, whereas focal ischemia causes a reduction of blood flow in a very distinct, specific brain region [202]. Changes in the B-CSF barrier after ischemic injury depends on the extent of anastomoses between supplying arteries, severity of the obstruction of blood flow and the duration of ischemia [203, 204].

Global cerebral ischemia induced by occlusion of four vessels led to CP cell necrosis after 6 h from reperfusion [204]. Similarly, necrotic CP epithelial cells, early damage to the organelles, plasma membrane and clumping of nuclear chromatin were found in the two-vessel model of forebrain ischemia with hypotension [205, 206]. Overall severity of ischemic insult is lower in the model of focal cerebral ischemia using middle cerebral artery occlusion as was proved by finding apoptotic cells characterized by nuclear DNA fragmentation only in the CP of the lateral ventricle in the ischemic hemisphere 6 h after middle cerebral artery occlusion [201]. MRI examination of B-CSF barrier integrity showed accumulation of contrast medium in the CP and the wall of the lateral ventricle on the ischemic side 6 h after middle cerebral artery occlusion. This was probably caused by apoptosis in the CP and leakage of contrast medium through the altered B-CSF barrier [207]. In both the models described above—global and focal cerebral ischemia 24 h after reperfusion—CP cells showed near-normal morphology, emphasizing the importance of CP for brain homeostasis [206, 208].

In contrast to morphological changes, molecular changes in the CP have a longer duration following ischemic insult. This is supported by decreased expression of AQP1 in CP epithelial cells up to 24 h, probably as a result of necrosis after global cerebral ischemia, but between 24 and 48 h the expression of AQP1 expression increased. This phenomenon could be explained by CP epithelial cell replenishment and restoration of the B-CSF barrier [209]. Interestingly, a more prolonged healing of the CP following global cerebral ischemia using the bilateral common carotid occlusion model was reported. These changes are related to CP endothelial cells that are detectable up to 7 days after the induction of global cerebral ischemia [210].

Ischemic injury of the CP led to increased permeability of B-CSF barrier for inulin 30 min after bilateral carotid occlusion at 6 h of reperfusion proving the effect of CP ischemic injury on B-CSF barrier function [205, 211]. Moreover, alteration in B-CSF barrier with calcium influx to CSF was observed up to 4 days following ischemic insult [210].

In the unilateral model of cerebral ischemia by occlusion of middle cerebral artery, reduced blood flow resulted in edema, apoptosis of CP epithelial cells, and alteration of B-CSF barrier only in the ipsilateral CP [207, 212]. An important factor in edema formation may be vasopressin and its receptor V1a which are expressed in CP blood vessels [213]. A similar finding with intense vacuolization (swelling), indistinct epithelial membranes, varying degrees of pyknosis was described in the middle cerebral artery occlusion model. In this case, MMP-9 level in the CP was increased as well, which can affect the integrity of B-CSF barrier [214].

In contrast to the transient forebrain ischemia and global cerebral ischemia models described above, proliferation and differentiation of CP epithelial cells was found 2 h after middle cerebral artery occlusion. This suggests that CP epithelial cells have also neural stem cell characteristics with the ability to express neuronal nuclear protein (NeuN) and glial fibrillary acid protein (GFAP) after mild to severe ischemia [215]. The mechanism by which the B-CSF barrier is stabilized is probably based on physical displacement of CP epithelial cells along the basal lamina. It was suggested that viable CP epithelium may “fill in gaps” caused by necrotic cells that undergo compression to render a full complement of cells covering each villus [206]. CP repair processes are probably accelerated by autocrine and paracrine mechanisms including expression of TGFβ1, BDNF and other growth factors [216]. Cytoplasmic fragments released from damaged epithelial cells following hypoxic exposure probably induces an immunological response with increased number of ED1+ macrophages, upregulation of C3 receptors and increased expression of MHC I and MHC II antigens. These pro-inflammatory changes are enhanced by overexpression of vascular endothelial growth factor (VEGF), eNOS and iNOS, which promote the adhesion of leukocytes to vascular walls [217, 218]. It was proposed that CP plays a major role as the invasion route for T-cells into the ischemic brain following a stroke. The mechanism of migration is probably based on a potential chemokine gradient between the CP and the cortical ischemic lesion [219]. A similar mechanism was found in monocyte-derived macrophages that were increased in the CP and CSF during the first week after ischemic insult. Gene expression of adhesion molecules and chemokines like VCAM-1, MAdCAM-1, C3CL1, and Nt5e were transiently upregulated during the same time period [220].

CP epithelial cells produce neurotoxic and inflammatory molecules after ischemic insult. These harmful substances can cause neuronal damage, especially in CA1 pyramidal neurons in the hippocampus [221]. However, there is evidence for a neuroprotective effect of transplanted CP after stroke. It has been found that transplanted CP induces secretion of growth factors including GDNF, BDNF, and NGF. This could be another therapeutic option after ischemic stroke or other neurological disorders [222–224].

## Reaction of the CP to nervous system trauma

Nervous system trauma leads to permanent or temporary impairment of the cognitive, physical, and psychosocial function of the nervous system. Moreover, it may result in neuropathic pain, which is characterized by hypersensitivity and ongoing spontaneous pain and is associated with activation of microglial cells and astrocytes.

There is a growing body of evidence indicating a robust B-CSF barrier reaction following traumatic brain injury (TBI) that originates as an external force acting on brain tissue (Table 2). The initial phase, known as primary injury, involves mechanical damage acquired at the moment of trauma. The CP of rats exposed to a non-penetrative impact developed ultrastructural changes expressed as widening of the intercellular spaces between adjacent epithelial cells [225]. Such widened spaces are likely to be involved in enhanced transepithelial transport of materials and secretion into the CSF. A breakdown of the B-CSF barrier induced by TBI was confirmed by uptake of serum albumin by CP epithelial cells [226]. Furthermore, increased numbers of macrophages in the epiplexus position associated with widened spaces between the CP epithelial cells were found after non-penetrative injury [227]. This surplus of epiplexal cells is related to increased levels of cytokines and chemokines in the CP [228]. It was also found that TBI in humans is associated with elevated levels of cytokines and chemokines in microdialysis perfusates [229]. In addition, elevated levels of the chemokine CCL2 was detected in the CSF of patients. An experimental mice model of TBI confirmed increased levels of CCL2 in the CSF along with a better TBI outcome in CCL2 knock-out mice pointing to the deleterious effect of this chemokine [230]. Hemodynamic changes following primary injury induce secondary damage of the CP that is similar to ischemic stroke.

In contrast to TBI, little is known about the response of CP to peripheral nerve and the spinal cord injury. The CP plays an important role in M2 macrophage trafficking to the injured spinal cord. VCAM-1-VLA4 adhesion molecules as well as epithelial CD73 enzyme play a major role in the extravasation and epithelial transmigration of M2 macrophages through the CP [231]. We observed increased numbers of ED1+ and ED2+ macrophages in the CP after peripheral nerve injury (Table 2; [232].

## Role of the CP in neurodegenerative diseases

Accumulating evidence is pointing out that the CP displays morphological and molecular changes that play a role in the progression and pathophysiology of the neurodegenerative disorders (Table 3). The most common neurodegenerative disorders and their relation to the B-CSF barrier are discussed below.

**Table 3:** Table summarizing changes of the CP in neurodegenerative and autoimmune diseases

Category	Disorder	Choroidal epithelial cells	Junctions	Immune cells	Transporters	Others
Neurodegenerative diseases	Alzheimer’s	↑ Lipofuscin vacuoles [301]	↓ Claudin-5, Claudin-1, Occludin, ZO-1 expression [233]		↓ AQP 1 expression [252]	Stromal fibrosis Thickening of blood vessel [249]
		↓ Mitochondrial activity ↑ Oxidative stress [246]			↓ Megalin expression [239]	Basement membrane thickening [249]
		↑ IL-1, IL-6, TNFα secretion MMP-3 expression [233]			↑ LRP1 and PgP expression [240]	↓ CSF production [248]
		CP epithelial cuboidal shape changed ↓ Nucleus size [233]			↓ TTR expression [244]	↑ Concentration of IgG [235]
		↑ IFN-I signaling ↓ IFN-γ signaling [236]				
		CP epithelial cell atrophy [241]				
	Parkinson’s				Modulation of transporters of alpha-synuclein [257]	
	Huntington’s		↓ Claudin-5 expression [258]			
Autoimmune diseases	Multiple sclerosis (MS)	↑ Cadherin expression [258]	↑ miRNA-155, miRNA-326 expression resulting in loss of TJs [268, 269]	↑ Lymphocytes in the vessels and stroma followed by increased IgG antibodies [260]		↑ C3d, C9neo expression within the pericapillary space in NMO [271]
		↑ CCR6 expression on Th-17 cells [265]				
		↑ CX3CL1 expression helping Entry of lymphocytes [267]				
		Swelling of CP [270]				
		↑ VCAM-1 and ICAM-1expression following increase in IFN-γ production [262]				
		↑ CCL5, CXCL9, CXCL10, CXCL11 expression upon peripheral immune stimuli [266]				
	ALS			Leukocyte trafficking through CP requires IFN-y signaling [272]		

### Alzheimer’s disease

Alzheimer´s disease (AD) is the most common neurodegenerative disorder characterized by dementia, synaptic loss and cognitive impairment. Overproduction of β-amyloid and subsequent deposits of β-amyloid plaques in brain tissue is the principal contributor to the pathogenesis of AD. It is becoming increasingly clear that CP dysfunction could be a major contributing factor to AD pathogenesis [233, 234].

It has been shown that the β-amyloid accumulated in the CP has an impact on the CP morphology and function. Presence of the β-amyloid in the CP is associated with immune responses mediated by innate immune reactions and increasing concentration of IgG that leads to capillary damage and interstitial fibrosis [235]. Expression of type I and II interferons involved in the recruitment of immune cells to the CNS was shown to be altered in the CP in an AD animal model [236, 237]. It has been found that β-amyloid induces increased secretion of pro-inflammatory cytokines (IL-1, IL-6, TNF-α) and matrix metalloproteinases (MMP-3) that have an impact on downregulation of TJ proteins (ZO-1, Claudin-1, Claudin -5, occludin) resulting in B-CSF barrier alteration in the early stages of the AD [233].

The CP and B-CSF barrier is involved in β-amyloid clearance from brain tissue. It has been shown that megalin, a multi-ligand endocytic receptor, is involved in the clearance of β-amyloid by transporting β-amyloid from CSF through the B-CSF barrier [238]. The level of soluble megalin secreted from the CP epithelium decreases in CSF in AD patients suggesting a possible contribution to β-amyloid accumulation in brain tissue [239]. β-amyloid accumulation also occurs via increased expression of transporter proteins such as PgP and low-density lipoprotein receptor-related protein 1 [11, 240–243]. Transthyretin (TTR) secreted by the CP has been described as the major β-amyloid binding protein in CSF. It plays a significant role in the clearance of excess β-amyloid from CSF and can inhibit β-amyloid aggregation and toxicity. Decreased expression of TTR in CP epithelial cells has been found in AD mice [244]. However, the level of TTR in CSF of AD patients is in the normal range [245]. Apart from the altered levels of transport proteins, mitochondrial activity deficit, oxidative stress, and morphological/structural changes in the CP also contribute to the decreased efficacy of β-amyloid clearance in the CP [234, 246].

Morphological changes in AD include atrophy of CP epithelial cells with accumulation of lipofuscin vacuoles, stromal fibrosis, and thickening of blood vessel walls and the basement membrane of the CP [247–249]. These morphological and molecular changes in the CP contribute to impairment of the B-CSF barrier that results in alteration of CSF composition in the AD brain [250, 251]. It has been described that CSF production is altered in AD probably due to thickening of the basement membrane, atrophy of CP epithelial cells [241] and decreased synthetic capacity of essential proteins for CSF production, such as carbonic anhydrase (CA) II and AQPI [233, 252]. In addition, expression of ion transporters involved in CSF secretion is decreased in AD [253].

Recently, it has been described that viral vectors targeting synthesis of β-amyloid in the CP could be useful in treating AD [254]. In addition, transplantation of CP epithelial cells has a curative effect via β-amyloid degradation [255].

### Parkinson’s disease

Little is known about the role of the B-CSF barrier in regulating alpha-synuclein in the CSF as crucial hallmark of Parkinson’s disease. Alpha-synuclein is expressed in the rat CP, primary culture of CP cells and an immortalized line of rat CP epithelial cells (Z310). This indicates that the CP can transport alpha-synuclein between the blood and CSF [256, 257]. However, this transport mechanism is not fully understood and needs further examination.

### Huntington’s disease

Analysis of post mortem samples of patients with Huntington’s disease revealed that cadherin was upregulated while claudin-5 was down-regulated implicating them in regulating the number of KC. Moreover, several neuroimmune-modulating interferons were significantly enriched [258]. Transplanted encapsulated CP has been shown to have a neuroprotective effect in Huntington’s disease [259].

## Role of the CP in autoimmune disorders of the CNS

Most CP changes in autoimmune disorders of the nervous system comes from studies on multiple sclerosis (MS), but little is known about the role of the CP in amyotrophic lateral sclerosis (ALS; Table 3).

### Multiple sclerosis

The MS is characterized by multiple inflammatory lesions of the white matter accompanied by an increase in T- & B-lymphocytes and macrophages. Inflammatory cells migrate through the brain barriers towards the CNS. Specifically, the CP is a site for lymphocyte entry to the CSF followed by increased synthesis of specific IgG antibodies [260]. In the CP of MS patients, lymphocytes were present in both the vessels and stroma. The presence of T-cells, especially CD4+ T-cells, was shown to regulate immune cell trafficking across the B-CSF barrier via the production of IFN-γ. Local IFN-γ signaling subsequently upregulates adhesion molecules such as VCAM-1 and ICAM-1 [71, 261–264]. Reboldi et al. [265] suggested that the migration of T-17 cells through the CP could be regulated by the chemokine receptor CCR-6. Recently, it was found that the CP upregulates chemokines (CCL5 and CXCL9–11) and cell adhesion molecules involved in T cell migration upon peripheral immune stimuli [266]. Other results showed increased levels of the chemokine CX3CL1 in the CP triggered by extracellular adenosine—a key mediator of lymphocyte entry into the CNS [267].

Up-regulation of microRNA-155 and -326 in MS is involved in the loss of TJ proteins leading to the disruption of B-CSF barrier. Subsequently, immune cells and autoantibodies penetrate into the CNS and induce inflammatory changes in oligodendrocytes [268, 269]. Murugesan et al. [270] investigated responses of the different CP compartments in the early stages of experimental autoimmune encephalomyelitis, an animal model for MS. They reported that the CP became swollen and the expression pattern of genes encoding adhesion molecules, interleukins and T-cell activation markers were different between capillaries and the CP epithelial cells. Inflammatory changes of the CP expressed as the presence of activated complement (C3d, C9neo) were also reported in neuromyelitis optica, a subtype of MS [235, 271].

### Amyotrophic lateral sclerosis

Local neuroinflammation contributes to the progression of ALS. IFN-γ signaling in the CP was reported to be involved in leukocyte trafficking during ALS progression [262, 272].

## Tumors of the CP

According to the 2016 World Health Organization (WHO) Classification of Tumors of the Central Nervous System, primary CP tumors are divided into CP papilloma (Grade I), atypical CP papilloma (Grade II) and CP carcinoma (Grade III; [273].

CP tumors vary from papilloma with cytological and architectural reproduction of normal CP to anaplastic and invasive neoplasms terminating in lesions lacking any morphological evidence of CP origin [274].

Several markers have been proposed to distinguish CP papilloma and carcinoma including VEGF, platelet-derived growth factor (PDGF) and its associated receptors PDGFRα, and PDGFRβ, but no reliable association has been found so far. On the other hand, Ki67 proliferative indices, the prevalence of glomeruloid microvascular proliferation and desmoplasia were found increased in CP carcinoma [275].

Moreover, expression of several proteins such as S-100, cytokeratin, vimentin and transthyretin (TTR) favors a CP papilloma diagnosis [276, 277]. Significant morphological differences were found between normal CP and CP papilloma, but not between CP papilloma and CP carcinoma. These changes include villous hypertrophy and diffuse enlargement of the CP with normal histological appearance [278]. Recently, it has been suggested that epigenetic alterations could be associated with specific CP tumor phenotypes. Hypermethylation in the promoter region of several genes like adenylate kinase, period circadian clock 2 (PER2), and phospholipid scramblase 4 (PLSCR4) leads to downregulation at the mRNA level that is typical for CP carcinoma. This epigenetic signature can distinguish aggressive forms of CP tumors from benign ones such as CP papilloma [279].

AQP1 may be another potential marker in discriminating between CP papilloma and CP carcinoma. The expression of AQP1 was found only in low-grade CP epithelial tumors, while high-grade CP carcinomas do not express this protein. This finding may be among the reasons why there are no described cases of hypersecretive hydrocephalus associated with high-grade CP tumors [280]. However, there is evidence for CSF overproduction and consequent hydrocephalus in low-grade CP tumors (especially papillomas) that can be explained by the enlarged CP surface [281, 282]. Interestingly, hydrocephalus was also described in CP neuroepithelial cysts. The authors suggest that increased numbers of water-filled vesicles in the CP can contribute to CSF overproduction and the consequent development of hydrocephalus [283].

To identify pathways involved in the pathogenesis of CP tumors, studies have focused on gene expression profiling. Twist-related protein 1 (TWIST1) is highly expressed in CP papillomas and involved in cell proliferation and invasion. This gene probably inhibits differentiation, interferes with the p53 tumor suppressor pathway and increases cell survival [284, 285]. The Myc proto-oncogene is a transcription factor, and its overexpression alone is sufficient to induce the development of CP carcinoma [286]. It has been found that overexpression of c-Myc in the CP epithelium leads to inflammation-dependent CP papillomas. The inflammatory infiltrate is composed of CD3+ T-lymphocytes (predominantly CD4+ T-helper cells) and CD68+ macrophages [287]. Expression of several markers like cytokeratin AE1/AE3, E-cadherin, N-cadherin, β-catenin and GFAP is indicative of primary CP tumors [288–290]. Despite bearing the general characteristics of primary CP tumors, they can be mistaken for metastatic carcinoma in biopsy samples (Table 4; [291].

**Table 4:** Table summarizing changes of the CP in tumors, schizophrenia and chronic stress

Category	Disorder	Choroidal epithelial cells	Immune cells	Transporters	Others
Tumors	CP papilloma	↑ c-Myc expression in CP epithelium [287]	↑ CD3+ T-lymphocytes, predominantly CD4+ T-helper and CD68+ macrophages [287]		Expression of S-100, cytokeratin, vimentin and TTR [276, 277]
					Villous hypertrophy and diffuse enlargement of the CP [278]
				↑ Expression of AQP1 [280]	↑ CP surface [282]
					↑ TWIST1 expression [284]
	CP carcinoma				↑ Ki67 proliferative indices [275]
					↑ Myc expression [286]
					Hypermethylation in the promoter region of AK1, PER2, PLSCR4 [279]
	CP cyst				↑ Water-filled vesicles number [283]
	Primary CP tumors				Expression of cytokeratin AE1/AE3, E-cadherin, N-cadherin, β-catenin, GFAP [288–290]
Schizophrenia		↑ Expression of genes related to immune function and inflammation associated with the disease status [296]			↑ CP volume [297]
Chorionic Stress		↓ 5HT2C, glucocorticoid receptor, cilia genes IFT88 expression ↑ 5HT2A, BDNF, TNFα, IL-1β expression [298]			

Several other rare neoplasms have been described in the CP, including B-cell lymphoma, T-cell lymphoma, malignant melanoma or fibrosarcoma [292–295].

## Effect of schizophrenia and chronic stress on CP

Increased CP volume and an associated upregulation of genes related to immune function in the CP has been observed in patients suffering from schizophrenia [296, 297].

Using proteomics and transcriptomics techniques, researchers have analyzed the changes in gene expression in the CP affected by chronic stress. They showed that 5HT2C, glucocorticoid receptor, and the cilia gene IFT88 were downregulated, while 5HT2A, BDNF, TNFa and IL-1b expression was upregulated in the CP [298].

## Concluding remarks

As we have described in this review, the pathogenesis and pathophysiology of various diseases is more or less linked with improper functioning of the CP and B-CSF barrier. We may conclude that the major changes are in the production of different proteins by CP epithelial cells, expression of TJ and transporter proteins as well as immune cell trafficking (summarized in the tables). However, it is questionable if the CP responds to different diseases in the same way in all ventricles. A comprehensive understanding of the role(s) played by the CP in different diseases would be the key that leads to new approaches to therapy. Currently, gene manipulation using viral vectors is on the rise and the CP would be a promising target for this sort of treatment.

Since CP epithelia have the ability to secrete different proteins into the CSF, viral vectors could be used to engineer CP epithelial cells to secrete therapeutic proteins or anti-inflammatory cytokines into the CSF in different neurological diseases. The importance of the CP is in forming the barrier between the blood and CSF to prevent the entry of harmful substances into the CNS. How this barrier functions, and in what way are the expression of TJ and transport proteins changed in different disorders still needs to be elucidated in detail. Nonetheless, it is clear that this barrier function can be used for treatment. Increased B-CSF barrier permeability would be useful for drug delivery, while on the other hand, closure of the barrier would be beneficial in preventing access to the CNS for pathogens or immune cells that can cause disease. Such barrier modulation can also be achieved using viral vectors. This underlines the absolute necessity for further studies in order to develop new therapeutic tools.

## Abbreviations

AJs: Adherent junctions
ALS: Amyotrophic lateral sclerosis
AQP: Aquaporin
BBB: Blood–brain barrier
B-CSF: Blood–cerebrospinal fluid
BVR: Biliverdin reductase
CNS: Central nervous system
CP: Choroid plexus
CSF: Cerebrospinal fluid
DAMPs: Damage-associated molecular patterns
EV: Extracellular vesicle
GFAP: Glial fibrillary acid protein
GJs: Gap junctions
Hamp: Hepcidin
HO: Heme-oxygenase
ICAM-1: Intercellular adhesion molecule 1
IVH: Intraventricular hemorrhage
KCs: Kolmer cells
LCN: Lipocalin
LRP1: Low-density lipoprotein receptor-related protein 1
LPS: Lipopolysaccharide
MAdCAM-1: Mucosal addressin cell adhesion molecule 1
MAPK: Mitogen-activated protein kinase
MCP-1: Monocyte chemoattractant protein-1
MMP: Matrix metalloproteinase
MRP: Multidrug resistance-related protein
MS: Multiple sclerosis
PgP: P-glycoprotein
TBI: Traumatic brain injury
TJs: Tight junctions
TLR: Toll-like receptor
TTR: Transthyretin
VCAM-1: Vascular cell adhesion molecule 1
VEGF: Vascular endothelial growth factor
ZO: Zonulin
